# A Case of Bright’s Disease of Mechanical Origin; Relief by Operation

**Published:** 1877-04

**Authors:** Samuel B. Ward


A Case of Bright's Disease of Mechanical Origin; Relief by Operation—*By Samuel B. Ward, A.M., M.D.*—Mrs. C. B., aged 58, American, widow, having a good family history in every way, contracted while residing in China, twenty-five years ago, a diarrhœa, which became chronic, and has had for years past from five to ten movements in the twenty-four hours, being frequently compelled to rise from one to three times at night.

On August 5, 1876, I was sent for to visit her on account of an acute aggravation of her bowel trouble, apparently dependent upon the fatigue incident to a trip to Philadelphia during the very hot weather. Finding that whatever she ate was passing undigested, lacto-peptine was ordered, and after three or four visits she expressed herself as so much relieved that attendance was discontinued.

On August 14th I was sent for again, and found that she had been very imprudent in her diet, and was much worse than when I first saw her—in fact, was seriously ill. The administration of twenty grains of calomel caused a very decided improvement in her condition within a few hours, and, after taking tonics and aids to digestion for a few days, her bowels returned to their habitual condition, and gave no further trouble.

One of the last days in August, at the request of a member of her family, she told me that she suffered severely, at times, with a neuralgia of the left shoulder; that she had curious sensations in her throat, such as led her to suspect that she might have a cancer there; that she had a good deal of dizziness and many unpleasant, indescribable sensations in her head; that she heard unnatural sounds in her ears; that she frequently saw a black cloud, and occasionally bright spots, floating before her eyes; and questioning concerning the urinary function brought out the facts that she was obliged to pass water about every two hours, night and day; that only half an ounce to an ounce was voided at a time; that passing this amount occupied from five to fifteen minutes, the urine escaping only drop by drop, while the act was accompanied by considerable effort and pain; and this condition of things began some years ago, and had been gradually growing worse. Subsequent examination of the urine showed it to contain albumen, the precipitate, after boiling and the addition of nitric acid, settling down to one-fourth or one-fifth of the bulk; the specific gravity was 1008; and the presence of hyaline and granular casts in large numbers was confirmed by the observation of my friend Dr. E. R. Hun. This, however, did not account for the length of time occupied in urination, and on September 1st Dr. James P. Boyd, Jr., saw the patient with me in consultation for the purpose of making a physical examination. We found the vagina so small as to admit the index finger only with difficulty; the uterus retroflexed and atrophied, with a well-marked conical cervix; and it was only after considerable search that we were able to find the orifice of the urethra, the meatus being covered over with a dense membrane, the opening in which would only admit an ordinary silver probe. Without much difficulty a director was substituted for the probe, and with a bistoury we made an incision downward and outward on each side, parallel with the ramus of the pubes, after which a full-sized gum-elastic catheter could readily be passed into the bladder. To prevent any recontraction, the catheter was passed every day for the first week, and every second day during the succeeding ten days, when its use was discontinued. After the trifling operation the patient experienced no delay in passing water when necessary, and no trouble in doing so except a slight burning pain during the first few days. Gradually she became able to retain her urine four or five hours durimg the day, and was not obliged to rise at night. The after-treatment was of the simplest possible kind, consisting of the administration of tonics—iron, quinine and strychnine.

The patient was quite weak for some days after the operation, but gradually gained strength, and, little by little, all the disagreeable symptoms on the part of the nervous system disappeared. Ten days after the operation chemical examination showed the urine to be free from albumen, and the microscope revealed only a few casts.

October 6th—just five weeks after the operation—it was not possible to find a single cast. The patient says that she feels better than at any time for four or five years past; is going all over the house at her pleasure; and that the diarrhœa of so many years' standing is also disappearing, the number of passages having been reduced to about two in the twenty-four hours.

November 10th.—Examination of the urine shows it to remain free from both albumen and casts. For two or three weeks past she has been going to church and about the streets in pretty much her usual health, the only remaining symptoms being that she occasionally has some slight unpleasant sensations in her head and shoulder. The condition of her bowels has so changed that she is occasionally obliged to take a laxative.

Remarks.—The interest in this case centres in the fact that the unpleasant and dangerous symptoms, on the part of the nervous system and intestinal tract, seem to have depended upon the Bright's disease. This latter, in turn, seems to have been of purely mechanical origin, depending upon the urethral obstruction. The violent contraction of the bladder which was necessary to overcome this, probably also forced back the urine through the ureters into the kidneys, and gave rise to the trouble which there existed. The diarrhœa appears to to have been only a compensation for the non-performance of their function by the kidneys. Certainly all the abnormal symptoms, some of them of many years' standing, almost entirely disappeared soon after the removal of the obstruction to the flow of urine.—*New York Medical Journal.*

